# Role of modified nasopharyngeal oxygen therapy in apnoeic oxygenation under general anaesthesia: a single-centre, randomized controlled clinical study

**DOI:** 10.1038/s41598-022-20717-3

**Published:** 2022-09-29

**Authors:** Weilian Geng, Changxing Chen, Yaobing Chen, Xinhua Yu, Shaoqiang Huang

**Affiliations:** 1 Department of Anaesthesia, Obstetrics and Gynecology Hospital, Fudan University, 128 Shenyang Road, Shanghai, China; 2 Department of Emergency and Critical Care Medicine, Shanghai General Hospital, Shanghai Jiaotong University School of Medicine, Shanghai, China; 3 The Division of Epidemiology, Biostatistics, and Environmental Health, School of Public Health, University of Memphis, Memphis, TN USA

**Keywords:** Medical research, Clinical trial design

## Abstract

Apnoeic oxygenation is not only important for patients who cannot be intubated/ventilated, but also can be routinely employed when planning to secure the airway.We aimed to compare safe apnoea times between patients receiving modified nasopharyngeal oxygen therapy and those receiving high-flow nasal oxygen therapy (HFNO) following the induction of general anaesthesia.This was a single-centre, randomized controlled clinical study. Eighty-four female patients undergoing elective laparoscopic gynaecological surgery under general anaesthesia were randomly assigned to the high-flow nasal oxygen therapy group (Group HFNO) or the modified nasopharyngeal oxygen therapy group (Group Naso). A Kaplan–Meier survival curve was used to describe the apnoeic oxygenation time.The safe apnoea time of the patients in the Group Naso was higher than that of the patients in the Group HFNO (20 (19.3 to 20.0) vs. 16.5 (12.9 to 20) minutes, P < 0.05). The incidence of SpO_2_ < 95% in the Group Naso was lower than that in the Group HFNO; hazard ratio 0.3 (95% confidence interval 0.2 to 0.6, P < 0.0001). Modified nasopharyngeal oxygen therapy which uses far less oxygen than HFNO is a convenient and effective method of apnoeic oxygenation in normal female patients.

Trial registration: https://www.chictr.org.cn, ChiCTR2000039433; date of registration: 28/10/2020.

## Introduction

During general anaesthesia, patients inevitably experience a period of apnoea from the start of anaesthesia induction to successful tracheal intubation. Even though the importance of preoxygenation with 100% oxygen has been pointed out in airway management for it can denitrogenates the functional residual capacity (FRC) of the lungs and hence increases the FRC oxygen store and delays the onset of arterial desaturation and hypoxemia^1–3^, inadequate preoxygenation is a common occurrence^4^.

During apnoea, the imbalance of carbon dioxide (CO_2_) and oxygen exchange results in negative pressure in the alveoli. This negative pressure in the alveoli is subatmospheric, generating a mass flow of gas from the pharynx to the alveoli, which is referred to as apnoeic oxygenation^5,6^. Apnoeic oxygenation, which can prolong the safe apnoea time, becomes particularly important for patients who potentially cannot be intubated/oxygenated after anaesthesia induction. Extending the safe apnoea time can provide opportunities for prolonged airway instrumentation and intubation.

High-flow nasal oxygen therapy (HFNO) is a recently developed apnoeic oxygenation technique^7^. It can effectively prolong the safe apnoea time of different groups of people^8,9^. However, HFNO is not sufficiently convenient for emergency cases of difficult intubation. Conventional nasopharyngeal airways also prolong safe apnoea time^10,11^, but they cannot directly supply oxygen. Therefore, few studies on apnoeic oxygenation are available.

**Figure 1:**
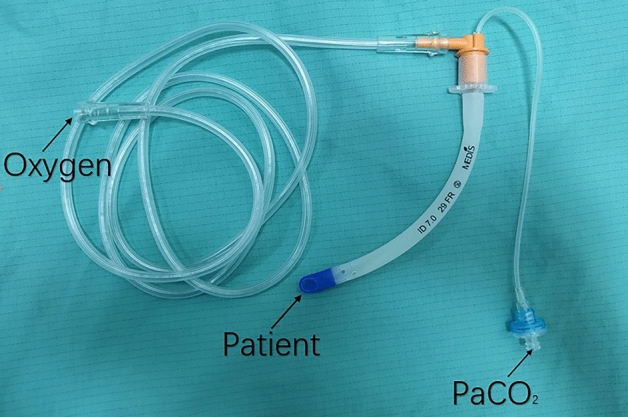
Modified nasopharyngeal airway with an oxygen catheter.

Compared with the traditional nasopharyngeal airway, a modified nasopharyngeal airway (Naso-Flo^®^ monitoring and filtration system, MEDIS Medical Tianjin, China) can be directly connected to oxygen (Fig. 1) and monitored end-tidal carbon dioxide (PetCO_2_) during mechanical ventilation. However, the use of modified nasopharyngeal airway is exactly the same as the traditional nasopharyngeal airway.Because the modified nasopharyngeal airway is a modified device, the patients who used the modified nasopharyngeal airway for oxygen therapy called modified nasopharyngeal oxygen therapy.We first studied the effectiveness of the modified nasopharyngeal oxygen therapy in prolonging the safe apnoea time of the general population. We hypothesized that a modified nasopharyngeal oxygen therapy would effectively and safely prolong the safe apnoea time.

## Results

### Characteristics of study subjects

**Figure 2:**
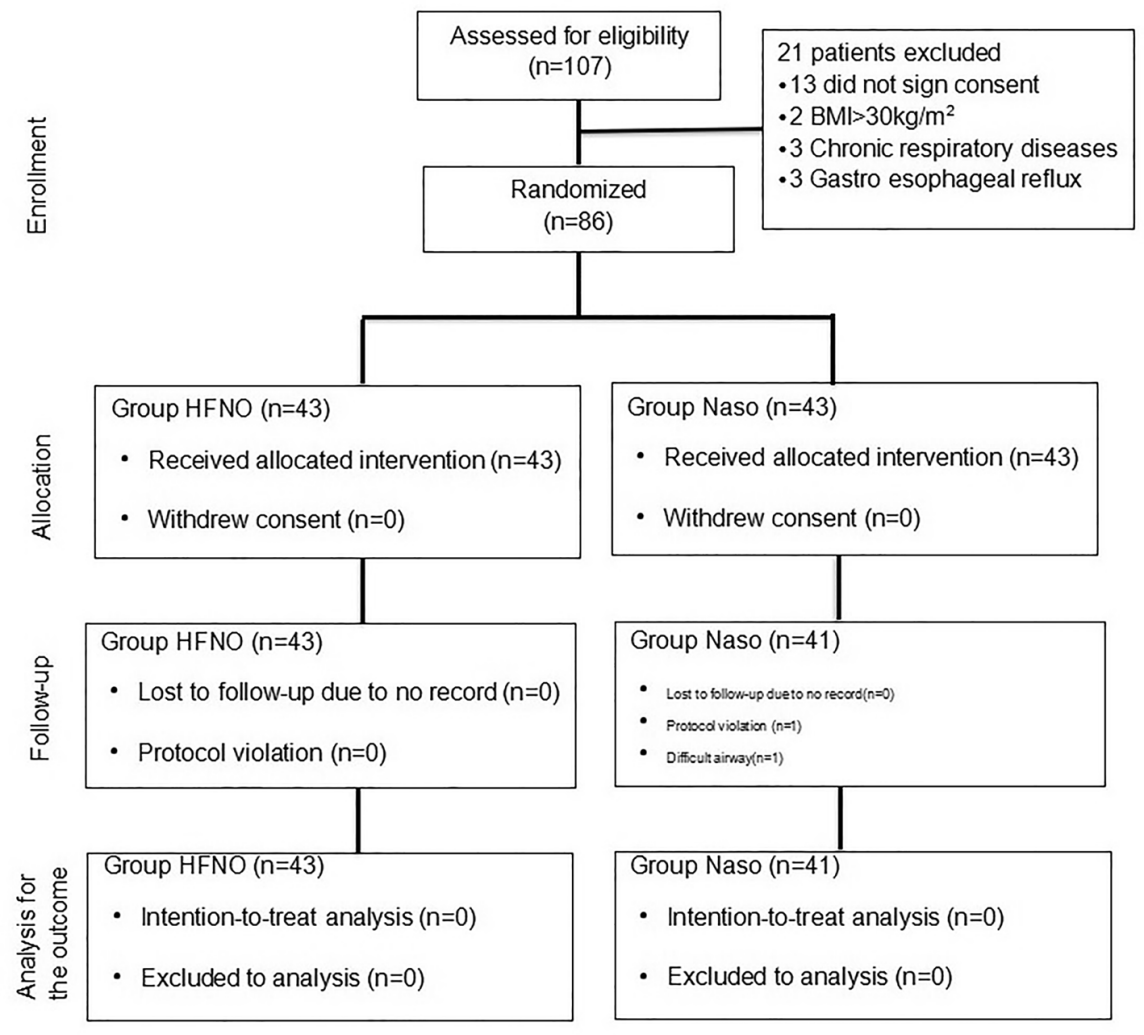
CONSORT flow chart.

A total of 107 female patients undergoing elective laparoscopic gynaecological surgery under general anaesthesia were included in this study. The final 86 patients were randomly divided into the Group Naso and the Group HFNO, with 43 cases in each group. Two patients in the Group Naso were excluded because the surgeon would not cooperate. Forty-three cases in the Group HFNO and 41 cases in the Group Naso were included in the final analysis (Fig. 2). The patients’ characteristics, basal SpO_2_, and FE’O_2_ between the two groups were not significantly different (Table 1).

**Table 1:** Baseline characteristics of included patients.

	Group HFNO (n = 43)	Group Naso (n = 41)	p valve
Age (years)	36.88 (10.43)	40.78 (10.36)	0.090
Height (cm)	161.35 (4.85)	160.10 (5.80)	0.286
Weight (kg)	56.83 (7.00)	58.09 (7.83)	0.439
BMI (kg/m^−2^)	22.08 (2.95)	22.58 (2.79)	0.425
Basic SpO_2_(%)	100 (100 to 100)	100 (100 to 100)	0.846
FE’O_2_(%)	93 (92, 94)	92 (92, 93)	0.279

Data are presented as mean ± SDs or mean (IQR).

### Primary outcome

**Figure 3:**
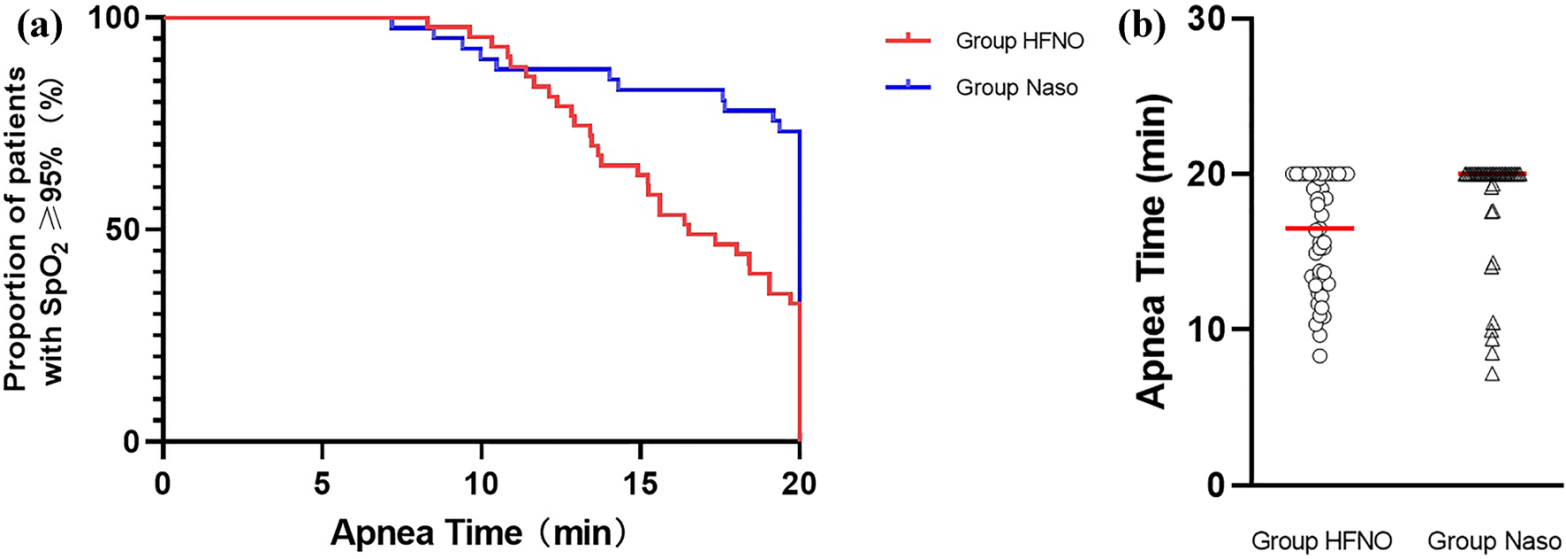
**(A)** Proportion of patients with SpO_2_ ≥ 95% during 20 min apnoea (HR 0.3, 95% CI 0.2 to 0.6, P < 0.001). (**B)** Safe apnoea time between the two groups, horizontal line = median. The safe apnoea time of the patients in the Group Naso was higher than that of the patients in the Group HFNO [20 (19.3 to 20.0) vs. 16.5 (12.9 to 20) min, P < 0.05].

At 20 min of apnoea, the ratios of patients with SpO_2_ ≥ 95% in the HFNO and Naso groups were 33% and 73%, respectively. Patients in the Group Naso were less likely to reach SpO_2_ < 95% in the first 20 min of apnoea; hazard ratio (HR) 0.3, 95% confidence interval (95% CI) 0.2 to 0.6, P < 0.001 (Fig. 3A). The safe apnoea time of the patients in the Group Naso was higher than that of the patients in the Group HFNO [20 (19.3 to 20.0) vs. 16.5 (12.9 to 20)] min, P < 0.05) (Fig. 3B).

### Secondary outcomes

**Figure 4:**
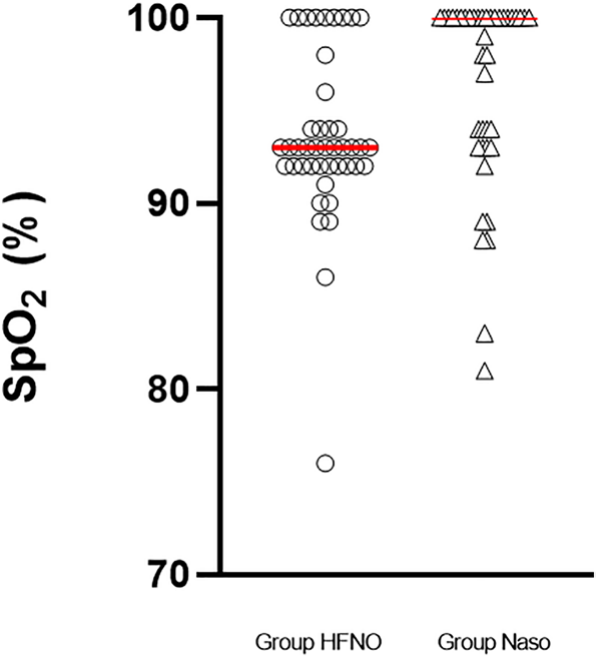
The lowest SpO_2_ between the two groups, horizontal line = median. The lowest SpO_2_ of the patients in the Group Naso was higher than that of the patients in the Group HFNO [100% (93% to 100%) vs. 93% (92% to 96%), P < 0.05].

**Figure 5:**
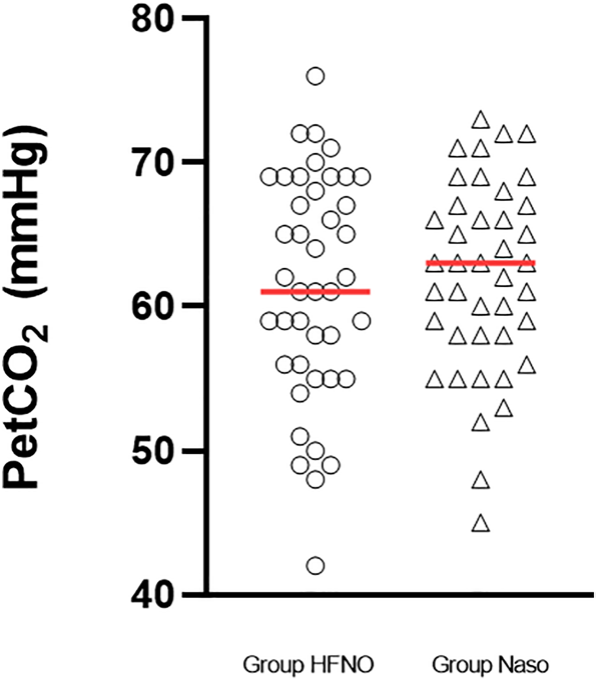
The first PetCO_2_ after mechanical ventilation between the two groups, horizontal line = mean. Group Naso was higher than that in the Group HFNO(60.9 ± 8.5 vs. 62.0 ± 6.6 mmHg, P < 0.001).

The lowest SpO_2_ of the patients in the Group Naso was higher than that of the patients in the Group HFNO (100% (93% to 100%) vs. 93% (92% to 96%), P < 0.05) (Fig. 4). After mechanical ventilation, the first PetCO_2_ measurement in the Group Naso was higher than that in the Group HFNO (60.9 ± 8.5 vs. 62.0 ± 6.6 mmHg, P < 0.001) (Fig. 5).

No significant differences in TcPCO_2_ per minute after apnoea were found between the two groups (P > 0.05). However, over time, the cases in the two groups gradually decreased. By 20 min, there were 14 cases in the Group HFNO and 30 cases in the Group Naso (P < 0.001).

Two patients in the Group Naso had hypotension events with a mean arterial pressure dropping to 69 mmHg and 66 mmHg after resuming mechanical ventilation. None of the patients in the Group HFNO experienced cardiovascular events such as hypotension (P = 0.152). There was no bleeding, pain or injury of the nose or nasopharynx in either group.

## Discussion

This study demonstrates a median safe apnoea time of 20 min in female patients receiving modified nasopharyngeal oxygen therapy. The results showed that the safe apnoea time of patients using a modified nasopharyngeal oxygen therapy in the study period were superior to those of patients undergoing HFNO. No patients complained of nasopharyngeal bleeding, pain or injury.

In past studies on HFNO, the safe apnoea time was 7.6–22.5 min^8,9,13–17^, which is similar to our results. Differing study populations and inconsistent study endpoints might have contributed to the inconsistency of research results.

Our study found that the safe apnoea time for patients using modified nasopharyngeal oxygen therapy was better than that for patients using HFNO^8,9,13–17^. The high-speed turbulence generated by high-flow oxygen in the upper respiratory tract causes Bernoulli effect, which conveys the air in the environment into the mouth. The higher the flow rate, the greater the turbulence, resulting in a higher degree of gas mixing in the mouth^18,19^. This may also be one of the reasons why the safe apnoea time of Group Naso was longer than that of Group HFNO because the opening end of the modified nasopharyngeal airway is closer to the trachea opening, it is less affected by the Bernoulli effect. This is also consistent with the viewpoint that the efficiency of apnoeic oxygenation was related to the distance between the location of the oxygen supply and alveoli, with a closer distance corresponding to higher efficiency^20^. The oxygen supply delivered through the nasopharyngeal airway opening is located at the oropharynx near the tracheal opening, while HFNO is administered through a nasal cannula, and the oxygen supply is located only in the nasal vestibule. Furthermore, compared with HFNO, this modified nasopharyngeal oxygen therapy can directly supply oxygen, which can reduce oxygen loss from the nasal cavity to the pharynx and improve the utilization rate of oxygen.

The biggest difficulty in our study is the setting of the end points. On the one hand, when we were designing the study, we looked through a large number of literatures and found that the research on apnoeic oxygenation can be roughly divided into two categories: one is that the end points of the study were artificially set, with a maximum apnoea time is 15 min^21^; The other is the study of apnoeic oxygenation for patients who cannot be intubated due to laryngeal and other operations. Its end points depend on the time of surgery, and the median apnoea time was 7.6–22.5 min^8,9,13–17^. Since our population is a relatively "healthy" population except for gynecological diseases, we speculate that it is possible to have a study end point ≥ 15 min. We hypoyhesized that the apnoea time of Group Naso was not lower than that of Group HFNO, so we finally chose 20 min as the end point of the study. We are also worried that the risk of hypoxemia and hypercapnia will increase with the extension of time, so the other endpoints of our study set as SpO_2_ falling to 95% or TcPCO_2_ ≥ 100 mmHg. When SpO_2_ continues to decrease, even if mechanical ventilation is restored, SpO_2_ decreases first and then increases, so the end point of SpO_2_ in our study was higher than the SpO_2_ decrease to 92% which would have been more clinically relevant in order to ensure patients’ safety. In addition, during the whole study, we closely monitored the patient's blood pressure, heart rate, SpO_2_, TcPCO_2_ and other vital signs to ensure the patients’ safety.

Even though the median apnoea time in the Group Naso was significantly longer than that in the Group HFNO by 4 min, the total range of apnoea times between the two groups was nearly identical, and the SpO_2_ of some patients dropped below 95% in less than 10 min in both groups,but there were more cases in Group Naso that could last for 20 min. Modified nasopharyngeal oxygen therapy also uses far less oxygen than the HFNO approach. From a resource utilization perspective, oxygen shortages have been reported related to a high burden of COVID patients in the ICU.

The apnoeic oxygenation technique prolongs the safe apnoea time after induction of general anaesthesia, which is accompanied by CO_2_ accumulation and hypercapnia. In this study, the participants were all ASA I-II patients undergoing gynaecological surgery, and arterial catheterization is usually not performed in such cases. Therefore, we did not routinely monitor PaCO_2_. Instead, for the safety of patients, we monitored TcPCO_2_ in real time through a transcutaneous CO_2_ monitor and one of the endpoits is TcPCO_2_ ≥ 100 mmHg . Our study found that CO_2_ accumulation was similar between the two techniques within the same period of apnoea. None of the patients in both groups had TcPCO_2_ greater than 100mmhg. However, with the extension of time, the number of cases in the two groups gradually decreased, especially in the Group HFNO, and only nine cases remained at 20 min. We still need further study of CO_2_ accumulation between the two techniques. The first PetCO_2_ in the Group Naso was higher than that in the Group HFNO, which was related to the longer safe apnoea time in the Group Naso.

Notably, patients in both the Group Naso and the Group HFNO had CO_2_ accumulation but did not show haemodynamic fluctuations due to hypercapnia. In the study by Frumin et al.^22^, the PaCO_2_ even reached as high as 160 mmHg, and the patients did not have serious side effects caused by hypercapnia. For patients who cannot be intubated/oxygenated after anaesthesia induction, the benefit of a prolonged safe apnoea time is far greater than the harm caused by CO_2_ accumulation. In addition, once ventilation is restored, accumulated CO_2_ can be rapidly controlled and effectively reduced. However, if the accumulated CO_2_ is removed too quickly, fluctuations in circulation may occur. Two patients in the Group Naso in this study developed hypotension after ventilation resumption. In addition, prolonged hypercapnia should be avoided as much as possible in patients who are intolerant of hypercapnia, such as those with elevated intracranial pressure, haemodynamic instability, and cardiac arrhythmias^7^.

This study has several limitations. First, the patients included in this study were all females between the ages of 18 and 65 years. Compared with females, males have a higher metabolic rate and oxygen consumption. Males also have different tracheal lengths and tracheal diameters^23^. Therefore, whether the inclusion of male patients would generate different results requires further study. Second, the present study included nonobese patients with a BMI < 30 kg/m^2^. Further studies are needed to investigate the role of modified nasopharyngeal oxygen therapy in apnoeic oxygenation in obese patients.

In summary, modified nasopharyngeal oxygen therapy which uses far less oxygen than HFNO is a convenient and effective method of apnoeic oxygenation in normal female patients. Future studies should focus on special patients, such as those with difficult airways and obesity, and on the role of modified nasopharyngeal oxygen therapy in prolonging safe apnoea time.

## Materials and methods

### Ethics approval and consent to participate

All procedures and protocols were implemented in accordance with the Declaration of Helsinki and the International Conference of Harmonization and Good Clinical Practice (ICH GCP). The study was approved by the Ethics Committee of Obstetrics and Gynecology Hospital of Fudan University (2020-149), and written informed consent was obtained from all participants. The trial was registered at https://www.chictr.org.cn (ChiCTR2000039433) on 28/10/2020 before patient enrolment. Trial reporting was in accordance with the 2010 CONSORT statement. The CONSORT flow diagram is shown in Fig. 2.

### Inclusion criteria

This study is a single-centre, randomized controlled clinical study. A total of 110 female patients aged 18–60 years with American Society of Anesthesiologists (ASA) grades I–II undergoing elective laparoscopic gynaecological surgery under general anaesthesia were enrolled in this study from November 2020 to July 2021. All enrolled patients signed informed consent forms for the study.

### Exclusion criteria

The exclusion criteria included the following: anatomical abnormalities or a surgical history of the face, nose, or upper airway; nasal obstruction or difficulty placing the nasopharyngeal airway; difficult airway; a New York Heart Association (NYHA) cardiac function classification > 2; smoking; chronic respiratory diseases; uncontrolled hypertension; elevated intracranial pressure; SpO_2_ below 97% after pure oxygen inhalation for 3 min; incorporation with facemask ventilation; a body mass index (BMI) ≥ 30 kg/m^2^; pregnancy; severe gastroesophageal reflux; neuromuscular disease; and refusal to participate in the study.

### Randomisation and blinding

Patients refrained from drinking and eating before surgery and did not receive any preoperative medication. After entering the operating room, the patients were randomly divided into the nasopharyngeal airway group (Group Naso) and the Group HFNO at a 1:1 ratio. An anaesthesia nurse who did not participate in the study prepared an opaque envelope containing grouping information in advance and numbered all the envelopes and the patients at the same time. Patient grouping was determined by a second random method based on computer-generated random numbers and the serial numbers of the opaque envelopes. Participants and outcome assessors were blinded to group allocation, while anaesthesia providers could not be blinded because of the significant differences between the anaesthetic techniques.

### Standard care and Interventions in both groups

Patients were placed in a supine position with their heads elevated approximately 15° and received a peripheral venous cannula. Electrocardiography (ECG), noninvasive blood pressure, and SpO_2_ were routinely monitored. In addition, a transcutaneous CO_2_ monitor (TCM400, Radiometer, Denmark) was connected until the transcutaneous carbon dioxide tension (TcPCO_2_) stabilized. The patient started facemask oxygen with an pure oxygen flow rate of 12 L/min. When the end-tidal oxygen fraction (FE’O_2_) was higher than 90%, intravenous injection of 2 mg/kg propofol and 0.5 µg/kg sufentanil was performed for anaesthesia induction. Rocuronium at a dose of 0.6 mg/kg was given intravenously, and timing was started.

Additionally, a nasopharyngeal airway or a high-flow nasal cannula was placed according to the group assignment. The patients in the Group Naso received a one-time sterile nasopharyngeal airway with an inner diameter of 6 mm or 7 mm according to whether the patient’s height was greater or less than 170 cm. One end of the nasopharyngeal airway was connected to oxygen, the oxygen flow rate was adjusted to 12 L/min, and the ratio of inspired oxygen was 100%. The patients in the Group HFNO inhaled warm humidified oxygen through the nasal cannula. The oxygen flow rate was 60 L/min, and the ratio of inspired oxygen was 100%. After 2 min of rocuronium administration, the patient underwent visual laryngoscopy to assess whether the airway was difficult to detect. If Cormack–Lehane grade was ≥ 3 under visual laryngoscopy, which indicates a difficult airway, the patient was removed from the study.

To ensure upper airway patency, jaw thrust was performed immediately, and the patient became unconscious and was maintained throughout the apnoeic period. The patients' mouths were open during the study.Furthermore, the SpO_2_ of the patient was closely monitored. If the SpO_2_ level dropped to 95% or the SpO_2_ level did not decrease to 95% but the safe apnoea time reached 20 min or TcPCO_2_ ≥ 100 mmHg, the timing was terminated. The attending anaesthetist proceeded with intubation under direct visualization through a video laryngoscope, followed by rapid reoxygenation with high tidal volume ventilation. Then, the oxygen concentration was adjusted to 100%, the tidal volume was 10 ml/kg, and the respiratory rate was 15 times/min to facilitate elimination of the accumulated CO_2_. The first partial pressure of end-tidal carbon dioxide (PetCO_2_) was recorded. When the patient’s PetCO_2_ was < 40 mmHg, the normal ventilation state was restored, and the study ended. 100% pure oxygen was inhaled during the study.

During the course of the study, propofol was continuously infused at a rate of 6 mg·kg^−1^·h^−1^ for anaesthesia maintenance, and the anaesthetic depth was adjusted according to a bispectral index (BIS) of 40–60. Changes in blood pressure and heart rate during the entire study period were recorded. Hypertension/hypotension was defined as an increase/decrease in the mean arterial pressure exceeding 30% of the baseline value, and a heart rate < 50 beats/min or > 100 beats/min was defined as bradycardia/tachycardia, respectively.

### Data recorded

The primary outcome was the safe apnoea time. The safe apnoea time was defined as the period from the patient’s respiratory arrest to a decrease in SpO_2_ to 95% or 20 min or TcPCO_2_ ≥ 100 mmHg. In this study, to reduce experimental errors caused by artificially determined respiratory arrest, based on previous literature, the start of apnoea (time zero) was defined as the time at which rocuronium was injected as a muscle relaxant^9,12^.

Secondary outcomes included the lowest SpO_2_, the time until the SpO_2_ returned to 100%, the TcPCO_2_ per minute after apnoea, and the first PetCO_2_ measurement upon resuming mechanical ventilation. The patient's age, height, weight, BMI, basal SpO_2_ on admission to the operating room, FE’O_2_ and SpO_2_ at the start of anaesthesia induction and the number of hypertension/hypotension and tachycardia/bradycardia events were also recorded. Pain, bleeding and injury to the nose and nasopharynx were also recorded when the patient was fully conscious .

### Statistical analysis

The sample size was determined based on the standard deviation of the two groups in the pre-experiment, which were 6.0 min and 5.9 min, respectively. We considered that the 2-min difference in safe apnoea time between the Group Naso and the Group HFNO had clinical significance. The α and β of the two-tailed test were set as 0.05 and 0.9, respectively, and 39 cases per group were required. Considering a 10% withdrawal rate, 43 cases were included in each group.

A Kaplan–Meier survival curve was used to describe the apnoeic oxygenation time. For measurement data such as the safe apnoea time, the lowest SpO_2_ and PetCO_2_ were analysed to determine whether the data were normally distributed. If the data were normally distributed, they were expressed as the mean ± standard deviation, and an independent samples t-test was conducted for intergroup comparisons; if the data were not normally distributed, they were expressed as the median (interquartile range (IQR) [range]), and the Mann–Whitney U test was used for intergroup comparisons. The incidence of cardiovascular events between these two groups was compared by the Chi-square test. Data were collated and analysed using SAS 9.4 (SAS Institute, Cary, NC). Graphs were plotted using GraphPad Prism 8.0. A p value of < 0.05 was considered statistically significant.

## Data Availability

The datasets and materials used and/or analysed during the current study available from the corresponding author on request.
